# Evaluating the Pathogenic Potential of IgE Targeting Cross-Reactive Carbohydrate Determinants in Dogs

**DOI:** 10.3390/ani14223275

**Published:** 2024-11-14

**Authors:** Thierry Olivry, Ana Mas Fontao, Laura Widorn, Ralf S. Mueller

**Affiliations:** 1 Nextmune AB, Riddargatan 19, SE 114-57 Stockholm, Sweden; 2 Nextmune Spain, Valentin Beato 24, 28037 Madrid, Spain; ana.mas@nextmune.com; 3 Center for Clinical Veterinary Medicine, Ludwig-Maximilians University Munich, 80539 Munich, Germany; l.widorn@medizinische-kleintierklinik.de

**Keywords:** allergy, dog, cross-reactive carbohydrate determinants, CCD, glycans, IgE

## Abstract

Cross-reactive carbohydrate determinants (CCDs) are complex N-glycans shared among allergens of plant, insect venom, and nematode origin. In allergic humans, IgE anti-CCD often develop and cause discrepancies between serological and skin tests. Overall, CCD-IgE are believed to be of low pathogenic relevance. IgE-targeting CCDs are also detected in companion animals, but their pathogenic potential and biological relevance are unknown. Herein, we first establish that, in 34 dogs with atopic dermatitis, the presence of serum anti-CCD IgE was detected in 14 pets (41.2%). In dogs, as in humans, IgE-targeting CCDs are heterogeneous, as they differentially recognized four distinct CCD-expressing proteins. The presence of CCD-IgE was associated with a higher and more frequent recognition of plant extracts in serological but not intradermal tests. Two different CCD-expressing proteins did not elicit immediate reactions when injected intradermally in dogs with detectable serum anti-CCD IgE. Similarly, two different CCD-expressing proteins did not induce the activation of mast cells passively transferred with canine anti-CCD IgE. Altogether, these results suggest that in dogs, as in humans, anti-CCD IgE are likely to have little pathogenic potential and blocking them in allergen-specific IgE serological tests is warranted to avoid false-positive results to plant extracts.

## Simple Summary

Cross-reactive carbohydrate determinants (CCDs) are complex sugars found mainly on plant allergens. In people with allergies, antibodies (IgE) against CCDs can develop, causing differences between blood tests and skin tests; these antibodies are believed not to be harmful. CCD-IgE are also found in pets, but their impact is unclear. In this study of 34 dogs with skin allergies, 14 dogs (41.2%) had detectable CCD-IgE antibodies. These antibodies reacted differently to four types of proteins with CCDs. Dogs with CCD-IgE showed more positive reactions to plant extracts in blood tests but not in skin tests. Injecting two CCD proteins into the skin of dogs with CCD-IgE in their blood did not cause immediate reactions. Similarly, these proteins did not activate mast cells, the main allergic cells. This suggests that, like in humans, CCD-IgE antibodies in dogs are not very harmful. Therefore, blocking these antibodies in allergy blood tests is important to help avoid false positives.

## 1. Introduction

**Figure 1:**
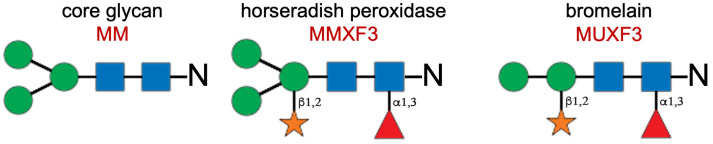
Major plant CCDs. Blue squares: N-acetyl glucosamines; green circles: mannoses; orange star: xylose; red triangle: fucose; N: asparagine.

Most allergens, especially those secreted, are glycan-carrying proteins or glycoproteins [1]. These glycans are either linked to asparagines (N-glycans) or threonines/serines (O-glycans), with most allergens carrying N-glycans and few being O-glycosylated [1]. The typical N-glycan shared among all eukaryotes is composed of a pentasaccharide core with two proximal N-acetylglucosamines and three distal mannoses (Figure 1 below) [1].

In the field of allergology, the term “*cross-reactive carbohydrate determinants (CCDs)*” has been used for decades to describe highly conserved, complex N-glycans added to plant (pollen, plant foods) and *Hymenoptera* venom allergens [2]. The “classical” plant CCDs have two crucial additions to the basic glycan core: a xylose with a β1,2 linkage to the most proximal mannose and a fucose attached with a α1,3 linkage to the proximal N-acetylglucosamine (Figure 1) [2,3]. Within the group of classical plant CCDs, there are two important ones. The first, abbreviated as MUXF3, only has two terminal mannose and is the main glycan carried by the pineapple enzyme bromelain [4]. The second, MMXF3, with three terminal mannoses, is typically present on the horseradish peroxidase (Figure 1).

As mammals have lost the ability to attach the β1,2 xylose and α1,3 fucose to their N-glycan cores, humans and animals can see these additions to plant N-glycans as foreign, leading to an abnormal anti-carbohydrate immune response [5].

Since first being reported in 1981 [6], anti-CCD IgE have been observed to occur naturally in a variable yet substantial fraction of allergic humans [7]. In such patients, the IgE seems to be directed principally against the α1,3 fucose [8,9], with the β1,2 xylose offering a secondary epitope [9]. In the last decade, anti-CCD IgE have also been reported in dogs [10,11,12,13,14,15,16,17], cats [14,18], and horses [19]. As in humans, anti-CCD IgE are detected principally in atopic dogs polysensitized to pollen, especially to grass pollen [10,12,14,15], as well as in dogs with high levels of IgE against *Hymenoptera* venom allergens (Nextmune PAX, unpublished data). CCD-targeting IgE can also be detected in healthy dogs [12].

There is ongoing debate regarding the pathogenicity of anti-CCD IgE in humans.

On the one hand, most of the evidence suggests low biological relevance for such IgE. For instance, several studies have demonstrated a difference between positive serological detection of IgE sensitizations to CCD-bearing plant allergens with corresponding negative skin prick tests to the same allergen extracts [20,21,22]. Additionally, the basophil activation test may be positive for CCD-expressing proteins, but only at concentrations multifold higher than those of plant allergen extracts to which the patients are truly allergic [20,23]. The sensitization to CCD-bearing allergens can result in positive allergen-specific IgE serology to allergens to which patients do not react clinically [20,22,24]. Finally, in five human patients with detectable IgE against rice-produced, CCD-expressing human lactoferrin (rhLF-CCD), a skin prick test with rhLF-CCD was negative, even though two individuals were shown to have a positive basophil histamine release with this protein [23]. In these five patients, a double-blinded, placebo-controlled oral challenge was negative with 1 g of rhLF-CCD [23]. This amount of ingested allergen is 10,000 times higher than that of the highest dose of recombinant Mal d 1 needed to induce flares in individuals with apple-induced oral allergy syndrome [25].

On the other hand, there is some evidence, albeit limited, of the possible pathogenicity and clinical relevance of anti-CCD IgE. For example, in a study of human patients allergic to olive tree pollen, it was discovered that two-thirds had IgE against Ole e 1’s single N-glycan. This glycan could induce a similar dose-dependent histamine release from basophils as the native allergen itself [26]. Another report included a single grass-allergic human patient with IgE only directed against the CCD; in this patient, Phl p 1 with only one glycosylation site failed to stimulate basophils, but Phl p 13, with multiple N-glycans, could do so [27]. In another study, some patients with tomato allergy had a positive basophil histamine release with the tomato extract and the fully glycosylated native Lyc e 2 (now Sola l 2) but not with the deglycosylated Sola l 2 [28]. These patients also had a basophil histamine release with clinically irrelevant CCD-expressing proteins, but not when these proteins were deglycosylated [28]. Likewise, individuals clinically allergic to *Cupressaceae* pollen had IgE that recognized the native but not the aglycosylated recombinant or the deglycosylated native Cup a 1; for these patients, it was the native, but not the recombinant allergen, that caused histamine release from basophils [29].

One of the first explanations historically proposed for the low pathogenic potential of anti-CCD IgE in humans was that some allergens, like Phl p 1 from Timothy grass, only had one N-glycosylation site carrying a complex glycan. Such a “CCD monovalence” would prevent allergens carrying single glycanic epitopes from cross-linking IgE on mast cells and basophils [27]. This hypothesis no longer seems valid, because, as mentioned above, many plant allergens (e.g., Phl p 13) carry several N-glycosylation sites with complex glycans [27,30]. In fact, the lack of pathogenic potential of anti-CCD IgE might be because they are “outcompeted” for binding to their target glycanic epitope(s) by anti-CCD IgG of much stronger affinity and higher serum concentration [31]. The daily consumption of plant-based foods is thought to be responsible for these elevated levels of neutralizing anti-CCD IgG in the bloodstream, a process of tolerization similar to what occurs during successful food oral immunotherapy [32,33].

Because of the existing postulation that CCD-IgE have little clinical relevance, strategies to “block” such IgE in serological tests were developed, usually by pre-incubating human sera with native, digested, or artificial CCD-expressing proteins unlikely to be sensitizing patients. Such inhibition of CCD-IgE results in a marked decrease in the sensitization rates to pollen, plant foods, and *Hymenoptera* venom extracts, as well as to native CCD-carrying molecular plant allergen components [34]. In veterinary allergology, the implementation of CCD IgE-blocking methods in serological tests is more recent [13,14]. As in humans, the incubation of sera with CCD-expressing proteins reduces the number of detected sensitizations to pollen extracts in dogs [13,14,15,16,17,35], cats [14,18], and horses [19].

There are currently no published studies evaluating the pathogenic potential of anti-CCD IgE in any animal species, despite the now common use of CCD-IgE blocking strategies in veterinary IgE serological tests. Our objective was thus to investigate whether CCD-expressing proteins could induce in vitro or in vivo mast cell degranulation, a proxy for their pathogenic relevance. We hypothesized that, like in humans, canine IgE directed against cross-reactive complex N-glycans are unable to activate mast cells and induce their degranulation. This incapacity would suggest their low pathogenic potential in dogs.

## 2. Materials and Methods

### 2.1. Study Subjects

We enrolled 34 dogs with atopic dermatitis (AD) diagnosed based on standard methods [36]; details of their signalment can be found in Supplementary Table S1. The breeds most often included were Labrador (5) and other retrievers (2), French bulldogs (4), and German shepherd dogs (3). They aged from 1 to 9 years (average: 4.0; median: 3.5 years), and the female-to-male ratio was 1.4. Twenty-eight of 34 dogs (82.4%) had signs that occurred during the entire year, three (8.8%) had seasonal signs, and the last three (8.8%) had year-long signs with a seasonal exacerbation.

Because interest had been expressed in pursuing allergen immunotherapy, intradermal testing (IDT) was performed, and blood was collected for allergen-specific IgE serology. The performance of these two interventions had been approved beforehand by the Ethics Committee of the Ludwig-Maximilians University of Munich; see the Institutional Review Board Statement at the end of this article.

### 2.2. ELISA

We used an enzyme-linked immunosorbent assay (ELISA) to determine the level of allergen-specific serum IgE against eight representative plant extracts. These included two extracts from grass pollen (Timothy grass *Phleum pratense*) and (Bermuda grass (*Cynodon dactylon*)), two from tree pollen (American sycamore (*Platanus occidentalis*) and olive tree (*Olea europaea*)), two from weed pollen (mugwort (*Artemisia vulgaris*) and wall pellitory (*Parietaria judaica*)), and the last two from plant foods (oat flour (*Avena sativa*) and carrot (*Daucus carotta*)). All extracts but two were purchased from Inmunotek (Madrid, Spain); the one from olive tree pollen was obtained from Stallergenes Greer (Lenoir, NC, USA), while that of oat flour was produced in-house by Nextmune (Madrid, Spain).

To detect the presence of CCD-specific IgE in the canine sera, we also performed ELISA using four different CCD-expressing proteins to which dogs are not usually sensitized. These proteins were purified bromelain from pineapple (*Ananas comosus*) (BRL; Ana c 2; B5144, Sigma-Aldrich, St. Louis, MO, USA), purified peroxidase from horseradish (*Armoracia rusticana*) (HRP; P3875, Sigma-Aldrich), recombinant human lactoferrin produced in rice (*Oryza sativa*) (rhLF-CCD; L4040, Sigma-Aldrich), and the MUXF3 complex glycan coupled to human serum albumin (HSA-CCD; Proglycan, Vienna, Austria). We also added purified HSA (SRP6182, Sigma-Aldrich) and hLF (L0520, Sigma-Aldrich) without CCDs to identify any canine sera with IgE directed against these human proteins. Altogether, these four distinct CCD-expressing proteins were selected because of their different numbers of N-glycosylation sites and type of complex N-glycan. Firstly, the synthetic CCD-HSA has eight to ten MUXF3 attached to the HSA (Proglycan, personal communication). In contrast, according to the N-glycosylation prediction algorithm NetNGlyc 1.0 (https://services.healthtech.dtu.dk/services/NetNGlyc-1.0/; page last accessed 12 October 2024), BRL has only one glycosylation site, which has been shown to carry MUXF3 as the main glycan [4]. Using the algorithm above, HRP is predicted to have nine glycosylation sites with eight (chain A) or seven (chain B) MMXF3 glycans. Finally, the rhLF-CCD is expected to have three glycosylation sites, with two of them shown to carry mainly MMXF3 or MUXF3 (~40% of each and 20% of other glycans) [37] (Figure 1).

Plates were coated with 1 µg per well of each allergen extract and CCD-expressing protein overnight at 4 °C. Following two washing steps with TBS/Tween 20 at 0.05% (TBST), 96-well plates were blocked with TBS/polyvinylpyrrolidone-10 (PVP-10) at 0.05% and then rewashed. Sera from the 34 dogs were incubated overnight on the plates at 1:10 in TBST. After further washing, we used alkaline-phosphatase-labeled 5.91 anti-canine IgE monoclonal antibodies (B. Hammerberg, NC State University, Raleigh, NC, USA) at 1 µg/mL in TBST/1% BSA [38]. After six washing steps, the chromogen para-nitrophenyl phosphate (pNPP, Moss, Pasadena, MD, USA) was applied for 30 min. The optical densities (OD) were read at 405 nm, and the threshold used for positivity was arbitrarily set at 0.150. All ELISA measurements were made in duplicates.

### 2.3. Intradermal Tests

Before performing the IDT, we ensured that, for each dog, the withdrawal times for drugs potentially interfering with this test were those recommended [39].

Our panel consisted of 39 allergen extracts (Nextmune, Lelystad, The Netherlands) that were injected intradermally. The concentration of the allergens used was 200 Noon Units (NU) for pollen antigens, 100 NU for mite extracts, 1000 NU/mL for the cat flea whole body extract, and 100 µg/mL for the *Malassezia pachydermatis* extract; the amount of allergen extract obtained from 1 g of raw material is defined as equivalent to 106 NU. Histamine phosphate and the dilution solution of the allergens (a phosphate-buffered saline solution with 0.47% phenol) served as positive and negative controls, respectively. If necessary, the dog was sedated with 0.04–0.08 mg/kg of dexmedetomidine (Dexdomitor, Zoetis GmbH, Berlin, Germany). There were 22 plant extracts with CCD-positive allergens: 5 from trees, 9 from grasses, and 8 from weeds (Supplementary Table S2).

In addition to the standard allergen extract panel above, we also wanted to test whether CCD-expressing proteins could induce mast cell degranulation in vivo. Thus, we injected the purified BRL and the hLF with and without CCDs at 200, 500 ng, and 1 µg per intradermal injection; these proteins are the same as described above for ELISA.

Fifteen and twenty-five minutes after allergen injection, each site was evaluated subjectively based on erythema, wheal size formation, and turgidity of the reaction, and the border of the wheal to the normal surrounding skin ranging; grades ranged from 0 (negative) to 4 (high reactivity), as reported previously [40]. For this study, we considered an intradermal reaction as positive when the subjective grading was >2 and when the wheal diameter was larger than the mean between the positive and negative control 15 min after injection.

### 2.4. Mast Cell Activation Test

To test if CCD bound to its target IgE could induce mast cell activation and degranulation in vitro, we used the recently developed mast cell activation test (MAT) that uses a conditional Hoxb8-immortalized mast cell progenitor line transgenic for the human high-affinity IgE receptor (FcεR1α) [41,42]. The extracellular segment of the human receptor has long been known to bind canine IgE and not IgG [43], thus confirming the validity of this heterospecific testing approach. Briefly, Hoxb8 mast cells were seeded in a 96-well round-bottom plate and passively sensitized overnight with 5 of the 34 canine sera described above. Then, the IgE-carrying mast cells were stimulated with 0, 1, 10, 100, or 1000 ng/mL of the same HSA-CCD, HSA, rhLF-CCD, and hLF used above along with an allophycocyanin (APC)-conjugated monoclonal antibody directed against mouse CD107a (LAMP-1) (1D4B, Biolegend, Amsterdam, The Netherlands). The CCD-HSA was chosen due to its multiple sites carrying only MUXF3, and rhLF-CCD was selected as it has two occupied glycosylation sites carrying either MUXF3 or MMXF3 or other minor glycans [37]. The stimulation with the proteins with or without glycans was performed for 30 min at 37 °C in the presence of 5% CO_2_. The percentage of activated mast cells was determined using flow cytometry based on the neo-expression of lysosomal CD017a on their membrane over the total number of acquired cells; this method has been described previously in greater detail [41,42]. As a positive control, we incubated the mast cells with canine sera, and instead of provocation with a CCD-expressing protein, we stimulated them with an antibody against the alpha subunit of the high-affinity IgE receptor (FcεRIα; CRA1, Miltenyi Biotec, Bergich Gladbach, Germany).

### 2.5. Statistics

We compared several parameters between dogs that had detectable IgE to CCD-expressing proteins (referred to as “CCD” dogs) and those that did not (referred to as “NCCD” dogs). The proportion of dogs with a positive ELISA or IDT to plant extracts was compared using Fisher’s exact test. The number of positive ELISA or IDT test results for plant extracts was compared using the Mann–Whitney test. Both tests were two-tailed and nonparametric, and the threshold for significance was set at 5%. Statistical comparisons were made using Prism 10.3.1 (GraphPad Software, Boston, MA, USA).

## 3. Results

### 3.1. ELISA

#### 3.1.1. ELISA with CCD-Expressing Proteins

**Figure 2:**
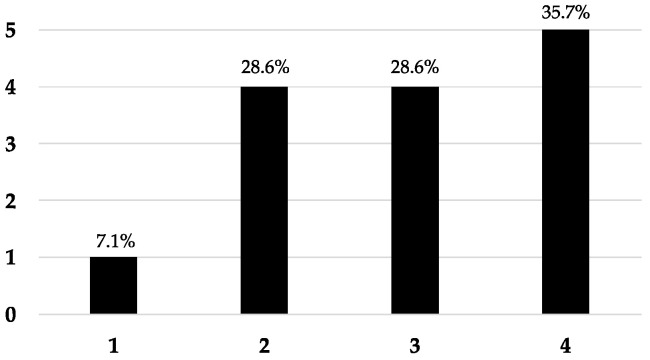
**Number of CCD-expressing proteins recognized in 14 dogs with AD (dogs CCD1-CCD14).** The x-axis represents the number of CCD proteins targeted by serum IgE, while the y-axis indicates the number of dogs recognizing that number.

We first tested the 34 canine sera by ELISA to search for IgE against the four CCD-expressing proteins (BRL, CCD-HSA, rhLF-CCD, and HRP) and their non-CCD-expressing controls hLF and HSA. Out of these sera, 14 dogs (41.2%) had detectable IgE to at least one of the CCD-expressing proteins, while none were found against the two controls (refer to Supplementary Table S3). We found that not all sera with detectable anti-CCD IgE reacted with all four proteins. In fact, these sera showed IgE recognition of between one and all four of these proteins in varying proportions (Figure 2). The CCD-expressing protein most often targeted was rhLF-CCD (13 dogs; 92.9%), followed, in decreasing proportion, by BRL (11 dogs; 78.6%), HSA-CCD (10 dogs; 71.4%), and finally HRP (7 dogs; 50.0%). In three dogs (CCD11, CCD12, and CCD13), the IgE detection of BRL and CCD-HSA was discordant, even though these two proteins are expected to carry the same MUXF3 glycan.

These results are consistent with a heterogeneity of epitopes targeted by CCD-IgE in dogs.

#### 3.1.2. ELISA with Plant Extracts

We then performed the same ELISA to detect IgE against eight extracts representative of the four main categories of CCD-expressing plant allergens: two each from tree, grass, and weed pollen and two from plant foods. All but two dogs (CCD3 and CCD12) with IgE against CCD-expressing proteins had detectable IgE against plant extracts (Supplementary Table S3). The number of extracts recognized varied between dogs from one to five of a maximum of eight tested (average: 1.9; median: 2.0); those of grass pollen were most often IgE-targeted (12/14 dogs; 85.7%). For dogs without detectable CCD-IgE, the corresponding values ranged from 0 to 2 (average: 0.2, median: 0.0).

**Figure 3:**
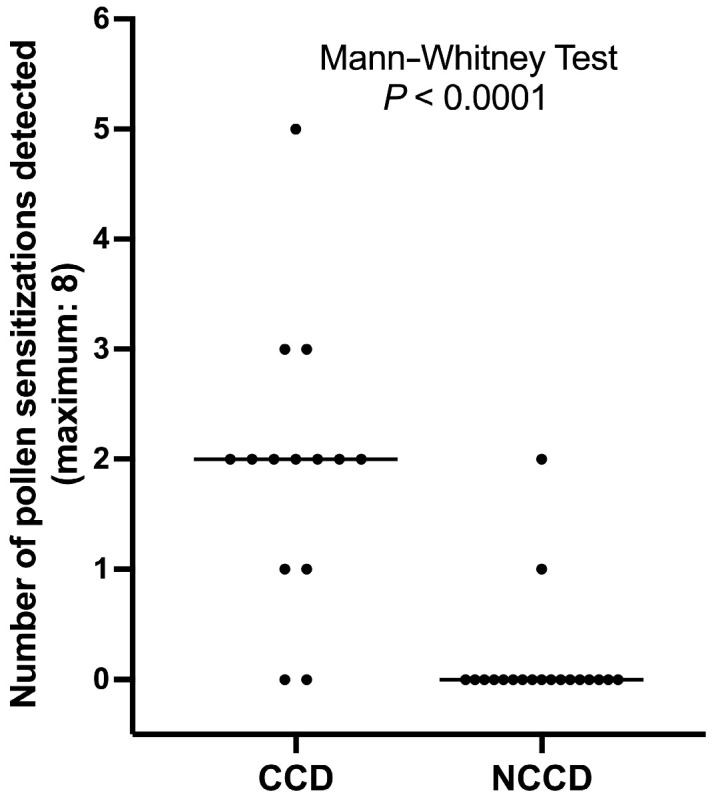
**Detection of serum IgE to plant extracts by ELISA.** Significantly more plant extracts were recognized in dogs with detectable CCD-IgE (CCD) than in dogs without such IgE (NCCD).

The number of dogs with positive IgE serology to plant extracts was significantly higher in dogs with positive ELISA to CCD-expressing proteins than in those with a negative ELISA against these proteins (Fisher’s exact test, *p* < 0.0001). Similarly, in dogs with detectable IgE against CCDs, the number of positive IgE to plant extracts was significantly higher than that of dogs without CCD-IgE (Mann–Whitney test, *p* < 0.0001; Figure 3).

The observation of a higher number of IgE sensitizations by ELISA in dogs with detectable CCD-IgE compared to those without is consistent with what has already been reported in allergic and normal dogs [10,12,13,17].

### 3.2. Intradermal Tests

#### 3.2.1. Intradermal Testing with CCD-Expressing Proteins

In dogs with anti-CCD IgE, intradermal injections of up to 1 µg of BRL and rhLF-CCD did not result in any positive reactions (Supplementary Table S3). In contrast, three dogs showed positive responses to the lowest (200 ng) or intermediate (500 ng) concentrations of hLF without CCDs. The absence of positive reactions to higher amounts of this protein (1 µg) and corresponding concentrations of the same protein with CCD (rhLF-CCD) suggests that these reactions were false positives.

Similarly, in dogs without detectable CCD-IgE, there were rare (12/178; 6.7%), inconsistent, positive reactions to intradermal injections of either BRL, rhLF-CCD, or hLF without CCDs (Supplementary Table S3). The lack of consistency (i.e., positivity at one concentration without reaction at higher ones) and the differences between hLF with and without CCD also suggest that these were also false-positive reactions.

Altogether, these results suggest that CCD-expressing proteins cannot activate mast cells loaded with CCD-IgE in vivo.

#### 3.2.2. Intradermal Testing with Pollen Extracts

Five of fourteen dogs (35.7%) with detectable serum CCD-specific IgE had positive IDT reactions to extracts from trees (1 dog; 7.1%), grasses (5 dogs; 35.7%), or weeds (2 dogs; 14.3%) (range of reactions: 0-6; average: 1.2; median: 0). In dogs without detectable CCD-IgE, there were four dogs (20.0%) with positive IDT to pollen extracts; these dogs reacted to grass (2 dogs; 10.0%) or weed pollen (3 dogs; 15.0%) (range: 0–7; average: 0.2; median: 0; Supplementary Table S3).

**Figure 4:**
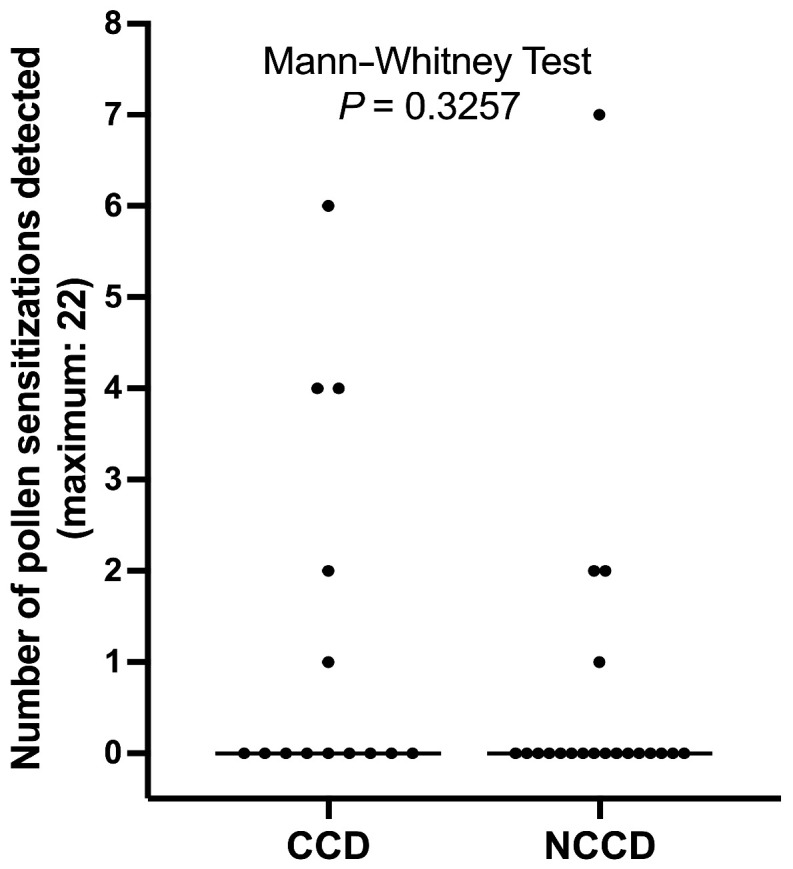
**Detection of reactions to plant extracts by IDT.** The number of positive responses to plant pollen was not significantly different between dogs with detectable serum CCD-specific IgE (CCD) and those without (NCCD).

The number of dogs with a positive IDT to plant pollen extracts was not significantly different between dogs with positive detection of IgE to CCD-expressing proteins than in those without such IgE (Fisher’s exact test, *p* = 0.4351). Similarly, the number of positive IDT reactions to pollen extracts was not significantly different between dogs with detectable serum CCD-IgE and those without (Mann–Whitney test, *p* < 0.3257; Figure 4).

Similar observations as those above have not been documented previously in dogs.

### 3.3. Mast Cell Activation Tests

**Figure 5:**
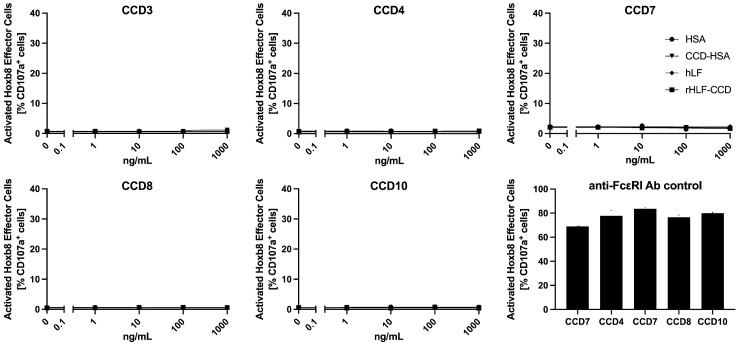
**Mast cell activation test with CCD-expressing proteins.** Five dogs with detectable serum CCD-IgE (refer to Supplementary Table S3 for their profiles) were passively transferred onto Hoxb8 mast cells transfected with the human high-affinity IgE receptors. There was no specific activation of mast cells after activation with either CCD-HSA, rhLF-CCD, or their non-CCD-expressing respective controls (HSA and rhLF). In contrast, there was a high percentage of activation when mast cells were incubated with each serum and stimulated with an antibody against the high-affinity IgE receptor FcεRI (bottom right).

To detect the ability of the CCD and its specific IgE pair to activate mast cells in vitro, we used the new activation test using Hoxb8 mast cells transfected with the human high-affinity IgE receptor, which is known to bind canine IgE [43]. After passive transfer with the sera of five dogs with detectable CCD-IgE, we stimulated the cells with five increasing concentrations (from 0.1 to 1000 ng/mL) of two CCD-expressing proteins or their non-CCD-carrying controls. Supplementary Figure S1 presents a composite of representative flow cytometry results for one of the five dogs tested (CCD4). As shown in Figure 5 below, neither of the CCD-expressing proteins led to a rise in the percentage of mast cells expressing the lysosomal protein CD107a, a proxy marker for their activation [41,42]. In contrast, when we incubated these mast cells with the same sera and an antibody against the high-affinity IgE receptor, between 60 and 80% of the cells expressed the activation marker CD107a (Figure S1).

These results suggest that sera containing CCD-IgE cannot stimulate mast cells in vitro even when challenged with CCD-expressing proteins carrying multiple complex glycans.

## 4. Discussion

To the best of our knowledge, this is the first report to evaluate the pathogenic relevance of CCD-targeting IgE in companion animal species. Our studies first revealed that the target of CCD-IgE varied from dog to dog. We also found that two single proteins expressing CCDs did not induce a positive IDT immediate reaction, even if the same dogs had detectable concomitant anti-CCD serum IgE. Finally, we confirmed that two CCD-expressing proteins did not activate mast cells after the passive transfer of dog CCD-IgE onto these cells. Altogether, these results suggest that CCD-targeting IgE have low pathogenic potential in dogs, as in humans.

The testing of four different CCD-expressing proteins by ELISA using the serum of 34 dogs with AD first revealed that 14 of them (41.2%) had detectable anti-CCD IgE in their serum, a percentage that is in the middle of the range (17% [12] to 73% [11]) previously reported in other studies that tested sera from allergic dogs.

This ELISA testing revealed that the anti-CCD IgE did not uniformly recognize all four CCD-expressing proteins. Instead, these IgE identified epitopes in between one and all four proteins. The most frequently identified protein was rhLF-CCD, while the least targeted was HRP. Our findings of CCD-IgE heterogeneity align with those of another study where sera from allergic dogs were tested for the detection of IgE against three different CCD-bearing proteins (BRL, HRP, and ascorbic oxidase); that study also observed different seropositivity rates to the various proteins [11]. This heterogeneity of reactivity to diverse CCD-expressing proteins suggests that, in dogs, as in humans [9,44], CCD-IgE likely recognize different epitope(s) on complex N-glycans. A logical hypothesis for the higher recognition of rhLF-CCD is that it has three predicted glycosylation sites, with two carrying both MMXF3 and MUXF3 types of complex plant N-glycans [37]. This complex glycosylation contrasts with that of BRL and CCD-HSA, which only have MUXF3, or that of HRP carrying MMXF3. Another surprise was the difference, in three dogs, in their IgE recognition of BRL and CCD-HSA, which are expected to both carry the same MUXF3. Even though the CCD-HSA has up to ten times more complex N-glycans than BRL, the slightly higher IgE binding to the latter perhaps resides in the presence of a second variant of MUXF3 that has three instead of the two usual terminal mannoses [4]. Finally, even though all tested proteins should have the same two characterized “CCD epitopes” for humans (i.e., those on the α1,3 fucose and β1,2 xylose [8,9]), our results indicate that the difference in external mannose between MUXF3 and MMXF3 seems to influence the recognition of CCD-IgE in dogs. An important consequence of the heterogeneity of CCD-IgE epitopes is that the successful blocking of circulating CCD-IgE in serological tests is expected to require a mixture of several proteins expressing different complex N-glycans rather than a single protein with just one type of carbohydrate.

The subsequent IgE serological testing of 34 sera using extracts representing different categories of pollen and plant foods showed the expected difference in the seroprevalence of reactivities between dogs that had CCD-IgE in their serum and those that did not. Indeed, as previously reported in dogs [10,12,13,17], the existence of CCD-IgE in the serum leads to a significant increase in serological reactivity to plant extracts, particularly to those of grass pollen. The presence of CCD-IgE is likely responsible for the observed increased number of positive results for pollen extracts, even though most dogs in that group exhibited perennial signs (Supplementary Table S1). The reason why not all pollen extracts tested positive in all dogs with CCD-IgE is puzzling. Still, this observation could reflect a variation in the number and amount of secreted CCD-expressing proteins in that extract. It is also surprising that, in this small series of dogs with CCD-IgE, two had negative IgE serology to all eight plant extracts tested despite one dog (CCD3) having high levels of IgE against two of the four CCD-expressing proteins and the second (CCD12) having low levels against two of the four (Supplementary Table S3). Altogether, the observed variation in plant extract reactivity in dogs with CCD-IgE likely indicates differences in epitope recognition by CCD-targeting IgE, as well as the variability and quantity of complex glycans expressed by allergens from different plant species.

This is the first report of IDT with CCD-expressing proteins in allergic dogs. The results were unequivocal: none of the 14 dogs with detectable CCD-IgE had a positive immediate reaction when injected intradermally with up to 1 µg of either BRL or rhLF-CCD. As this protein amount is known to be sufficient to elicit a positive skin test in an allergic dog [45,46,47], the lack of a positive reaction is, therefore, unlikely to be due to the quantity of protein injected being too small. Instead, it likely reflects the inability of CCDs to cause the in vivo degranulation of mast cells coated with CCD-IgE.

In contrast to the significant difference in the rate and number of positive ELISA results to eight plant extracts between dogs with or without CCD-IgE, there was no difference in the rate and number of positive IDT reactions to 22 plant extracts between the two groups of dogs. This difference in test results highlights the strong influence of CCD-IgE on serological but not skin tests. Despite this lack of influence of CCD-IgE, the results of IDT did not correlate at all with the seasonality of signs experienced by the patient. Indeed, the IDT was positive for pollen extracts only in 2/6 (33%) dogs with a seasonal worsening of signs. At the same time, the test was positive in seven dogs with perennial signs (Supplementary Tables S1 and S3). A similar lack of correspondence between IDT results and the seasonality of signs in allergic dogs has been reported recently [48].

In our last experiment, we tested the ability of CCDs to activate mast cells coated with CCD-IgE from five canine sera. We found that even at a concentration of 1000 ng/mL, which is 100 times higher than the concentration of allergens first inducing mast cell activation in human peanut-allergic sera [42], there was no mast cell activation when using two different CCD-expressing proteins with either eight to ten MUXF3 (CCD-HSA) or two glycosylation sites (rhLF-CCD) with MUXF3/MMF3 and minor glycans. Of note is that an allergen concentration of 100 ng/mL is sufficient for this test to detect pathogenic peanut-specific IgE in human sera with optimal sensitivity and specificity [42].

We identified three limitations in the current studies. First, some may argue that the number of dogs tested is relatively small to generalize our results to the entire population of allergic dogs. However, despite this small number, the breeds and ages of the dogs tested appeared to be representative of dogs with atopic dermatitis. Second, we noticed some inconsistencies in the ELISA testing of CCD-expressing proteins and only speculated about the reasons behind these differences. Finally, although we did not observe mast cell activation with CCD-expressing proteins in vivo or in vitro, we have not yet performed experiments to explain this lack of activation; such studies are forthcoming.

## 5. Conclusions

The studies mentioned herein provide evidence for the diversity of anti-CCD IgE in dogs as in humans. We also observed that injecting two CCD-expressing proteins intradermally did not cause immediate reactions. Additionally, two different CCD-expressing proteins did not activate mast cells in vitro after the passive transfer of canine sera containing anti-CCD IgE. Overall, these findings suggest that CCDs are unlikely to trigger allergic responses in dogs with detectable serum CCD-IgE, like in humans. Consequently, it is critical to use potent CCD blockers in IgE serological tests—and to verify the efficiency of such CCD blocking—to reduce the probability of false-positive results, especially for grass pollen. Due to the diversity of CCD-IgE, any effective CCD-blocking strategy will likely require a combination of CCD-expressing proteins.

## Data Availability

All data for these studies are included in Supplementary Table S3.

## Supplementary Materials

The following supporting information can be downloaded at https://www.mdpi.com/article/10.3390/ani14223275/s1. Table S1: Signalment of study subjects; Table S2: List of allergen extracts used for intradermal testing; Table S3: Intradermal and serological test results. Figure S1: Representative flow cytometry data during mast cell activation test.
