# Supplementary material for: Evaluating the Pathogenic Potential of IgE Targeting Cross-Reactive Carbohydrate Determinants in Dogs
Source: Animals (Basel). 2024 Nov 14;14(22):3275. doi: 10.3390/ani14223275 (PMC11591198; doi:10.3390/ani14223275)

Figure S1: representative flow cytometry data during mast cell activation test

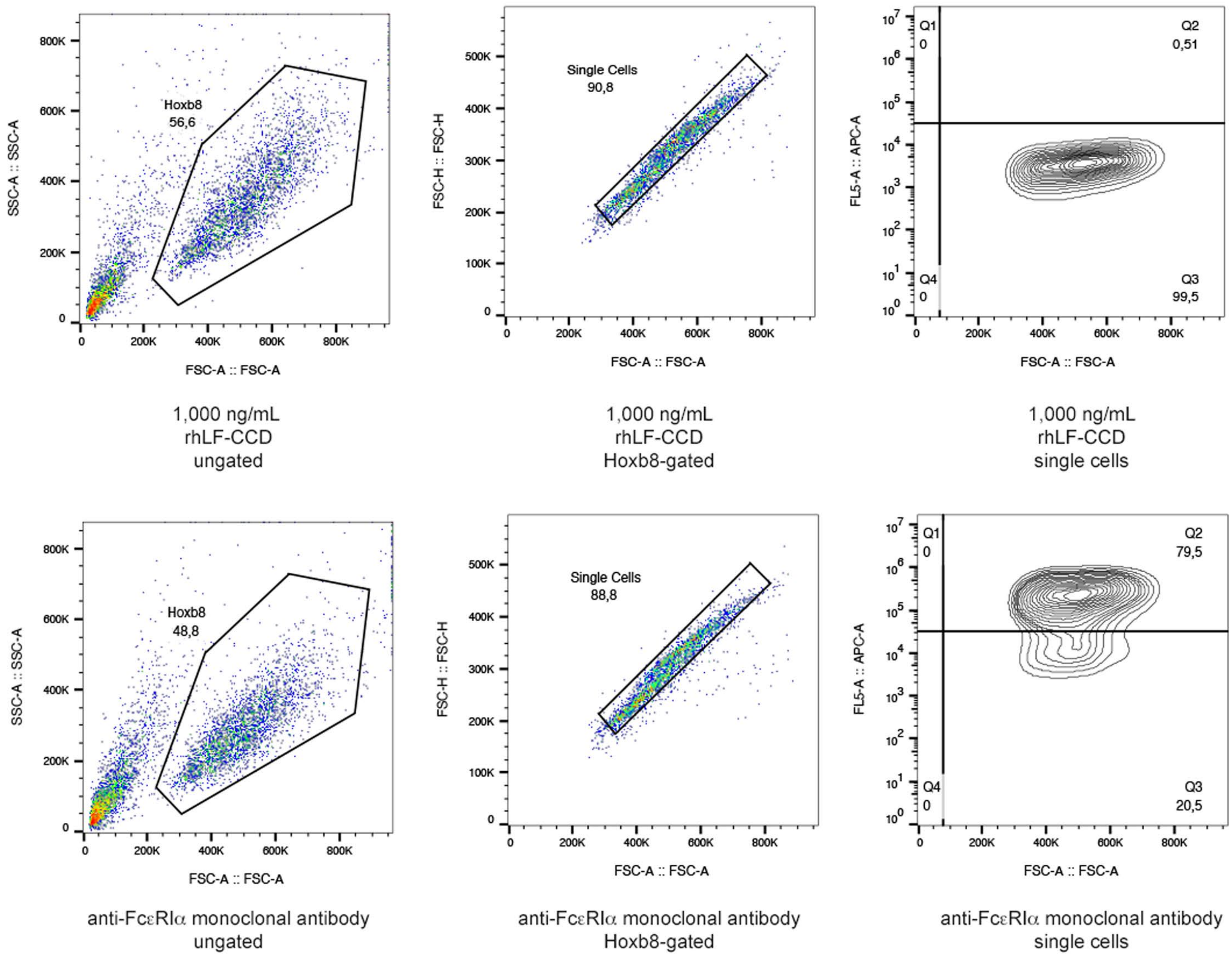

Supplement: Supplementary file 1 [file animals-14-03275-s001.zip › FigureS1_flowcytometry_MAT.pdf]
